# Fast-tracking fungal detection: a rapid flow cytometric method for the detection of yeasts in blood cultures

**DOI:** 10.3389/fmicb.2026.1813126

**Published:** 2026-04-23

**Authors:** Malgorzata K. Kopczyk, Kieran Mulroney, Graham Weaire-Buchanan, Teagan Paton, Emily Salisbury, Wai Shaun Ho, Dianne J. Gardam, Ammie Higgins, Christine F. Carson, Tim J.J Inglis, Aron Chakera

**Affiliations:** 1 School of Medicine, University of Western Australia, Perth, WA, Australia; 2 Harry Perkins Institute of Medical Research, Perth, WA, Australia; 3 PathWest Laboratory Medicine WA, Fiona Stanley Hospital, Perth, WA, Australia; 4 PathWest Laboratory Medicine WA, QEII Medical Centre, Perth, WA, Australia; 5 School of Pathology and Laboratory Medicine, University of Western Australia, Perth, WA, Australia; 6 Department of Renal Medicine, Sir Charles Gairdner Hospital, QEII Medical Centre, Hospital Avenue, Perth, WA, Australia

**Keywords:** blood culture, candidemia, detection, flow-cytometry, yeast

## Abstract

Invasive fungal diseases (IFDs), such as candidemia are increasing in prevalence globally and impose greater risk of life-threatening infection for immunocompromised and hospitalized patients if not treated with appropriate antifungals. The “gold-standard” method for candidemia detection is culture based with low sensitivity, often taking days to obtain results. We have developed a novel flow-cytometric method that detects yeast cells in blood cultures using two fluorescent markers, providing results in just over 1 h. In our study, we investigated the performance of the markers in clinical isolates across 6 different yeast species in spiked blood cultures. Using flow-cytometry and confocal microscopy we investigated the staining specificity of the markers to non-yeast cells (i.e., human cells and bacteria) and validated our gating strategy using imaging cytometry. Our limit of detection using conventional flow cytometry was 2.871 × 10^3^ cells/mL in spiked cultures and we successfully applied our assay to yeast positive clinical blood cultures, with the ability to detect unusual species such as *Saccharomyces cerevisae*. Compared to conventional microscopy, our assay demonstrates a superior LOD and with further optimization, could detect candidemia earlier to potentially improve patient outcomes.

## 1 Introduction

Invasive fungal diseases (IFDs) are associated with significant morbidity and mortality, affecting over 6.5 million people each year and causing an estimated 3.8 million deaths globally (Denning, 2024). IFDs are a significant burden to healthcare systems with an estimated cost of $7.2 billion in the US alone in 2017 (Benedict et al., 2019). These fungal infections threaten individuals with weakened immune systems including people with HIV/AIDS, cancer, chronic respiratory diseases, and patients with solid organ transplants (Sipsas and Kontoyiannis, 2012; Limper et al., 2017; Trelles et al., 2025). Studies have also suggested that the geographic range and incidence of IFDs is increasing due to climate

change, further deepening the impact of these diseases (Konkel Neabore, 2024). The World Health Organization (WHO) has recognized this growing threat and developed the WHO fungal priority pathogens list (WHO FPPL) which aims to drive additional research into these fungal pathogens and promote monitoring of antifungal resistance (WHO, 2022).

IFDs can cause systemic infections, and common causative agents include *Candida, Cryptococcus, Aspergillus*, and *Pneumocystis* species. Invasive candidiasis is a critical infection and is considered the most common IFD, particularly in hospitalized patients (Kullberg and Arendrup, 2015; Logan et al., 2020). Invasive candidiasis includes clinical conditions such as candidemia and deep-seated candidiasis (Logan et al., 2020). Candidemia is a serious blood stream infection with mortality rates ranging from 28% (Thompson Iii et al., 2024) to 40.4% (Salmanton-García et al., 2024). Candidemia infections are predominately caused by *C. albicans* (48%−51%), followed by *C. parapsilosis* (17%−28%) and *Nakaseomyces glabratus* (formerly *C. glabrata*) (7%−20.3%) (Tortorano et al., 2013; Mesini et al., 2020; Brescini et al., 2022). Other species such as *C. tropicalis* and *Pichia kudriavzevii* (formerly known as *C. krusei*) have been isolated from 9.3% and 2.8% of candidemia cases, respectively (Pfaller et al., 2019). Of increasing concern is the emergence and global spread of *C. auris*, a nosocomial multidrug resistant pathogen that is associated with high mortality rates (Zhang et al., 2025). Since the initial report of the pathogen in Japan in 2009 (Satoh et al., 2009), *C. auris* has spread in hospitals in Europe, the US, India, Pakistan and South Africa (Politi et al., 2024; Zhu et al., 2020; Ashraf et al., 2023; Farooqi et al., 2020; Shuping et al., 2023) with approximately 90% of isolates resistant to the most widely administered antifungal–fluconazole (Chowdhary et al., 2023). This emerging pathogen has been added to the critical priority group (along with *C. albicans, Cryptococcus neoformans* and *Aspergillus fumigatus*) in the WHO FPPL to highlight the need for urgent, focused research efforts (WHO, 2022).

The “gold-standard” for the diagnosis of candidemia is a positive blood culture which is conventionally performed in an automated system that measures metabolic products produced in incubated blood culture bottles (Slepčanová et al., 2025). This incubation step increases the concentration of yeast cells as initial blood culture samples in candidemia cases have a median of 1 CFU/mL or vary greatly in concentrations (0.1 to > 1,000 CFU/mL) (Pfeiffer et al., 2011). In the Bactec FX blood culture systems, a “positive” signal for growth generally requires 1.2 × 10^6^ – 8.5 × 10^6^ CFU/mL for yeast samples which are then examined microscopically and cultured for further analysis using routine phenotypic methods (Yan et al., 2011). This “gold-standard” method for detection has slow turnaround times (2–7 days for final results) and low sensitivities (positive result obtained in only 50%−75% of candidemia cases) (Clancy and Nguyen, 2013; Slepčanová et al., 2025). Delays in the initiation of appropriate antimicrobial treatment can have significant impacts on mortality rates (Garey et al., 2006; Morrell et al., 2005) and this has spurred the development of various rapid, culture independent tests including genotypic methods (DNA detection) and the testing of yeast biomarkers (e.g., 1,3-β-D-glucan). While PCR based detection methods are rapid (~1 h for results) and can be highly sensitive and specific (percentages of 95% and 92% respectively in suspected candidemia cases) (Avni et al., 2011), disadvantages include the inability to distinguish between live/dead organisms and the potential for false-positive results due to contamination (Salimnia et al., 2016; Slepčanová et al., 2025). In addition, these molecular based methods are limited to the detection of *Candida* species included in the test panel and can consequently fail to detect unusual species causing candidemia such as *C. dubliniensis* (Simor et al., 2018). Testing of 1,3-β-D-glucan in serum/plasma is also rapid (~1 h in automated systems), with sensitivity and specificity percentages of 69.9% and 87.1% reported in a multicenter evaluation study (Ostrosky-Zeichner et al., 2005). One main limitation of these bio-marker based assays is the risk of false-positives as numerous medical products can be contaminated with glucans and cause elevated 1,3-β-D-glucan levels in the absence of infection (Slepčanová et al., 2025). The implementation of these methods into the clinical laboratory can be limited by factors including availability and cost—particularly when using commercial products. While results for PCR and serum testing methods are available faster compared to conventional methods, they are typically used in combination with culture-based methods due to their limitations (Slepčanová et al., 2025).

Flow cytometry is already widely used in clinical labs to assist in the diagnosis of diseases including primary immunodeficiencies and hematologic malignancies. While flow cytometry has traditionally been focused on human cell profiling there has been renewed interest in using the technology for microbial cell detection in biological fluids (Rubio et al., 2019). Numerous studies have investigated the utility of flow cytometry for the detection of bacteria in samples including blood (Huang et al., 2019) and urine (de Boer et al., 2017), however there are few studies on yeast cell detection. In the case of detection in blood cultures, this may be attributed to difficulties in the processing of a complex sample matrix as lysis of erythrocytes can create excessive levels of background noise which can impair flow cytometric analyses (Huang et al., 2019). The high ratio of human cells compared to micro-organism cells, also makes detection more challenging (Pitt et al., 2016). In addition, biochemical similarities between yeast and human cells (Kachroo et al., 2022) can make the search for highly specific markers difficult. Despite these challenges, flow cytometry has numerous advantages over other phenotypic detection tests (such as microscopy) including rapid single cell profiling and the capacity for high-throughput sample analysis. Compared to genotypic methods, flow cytometry can reliably distinguish viable from non-viable organisms, detect sub-populations, and provide precise quantitative data of organism burden in clinical specimens (Álvarez-Barrientos et al., 2000; Śliwa-Dominiak et al., 2025).

To address the need for additional rapid diagnostic tests, we developed a method to detect and reliably enumerate yeast cells in blood cultures using flow cytometry. We investigated the staining specificity of three fluorescent markers: Calcofluor white (CFW), Concanavalin A (ConA), and Anti-*Candida* FITC (ACF) to yeast isolates from 6 clinically important species (including *C. auris*). Our final method consists of a positive blood culture clean-up step with samples dual stained with CFW and ACF—a combination of markers not used in previous studies. After staining, data are acquired on the Attune^TM^ NxT and the time to result from sample processing to data collection is just over 1 h.

## 2 Materials and methods

### 2.1 Study design

**Figure 1:**
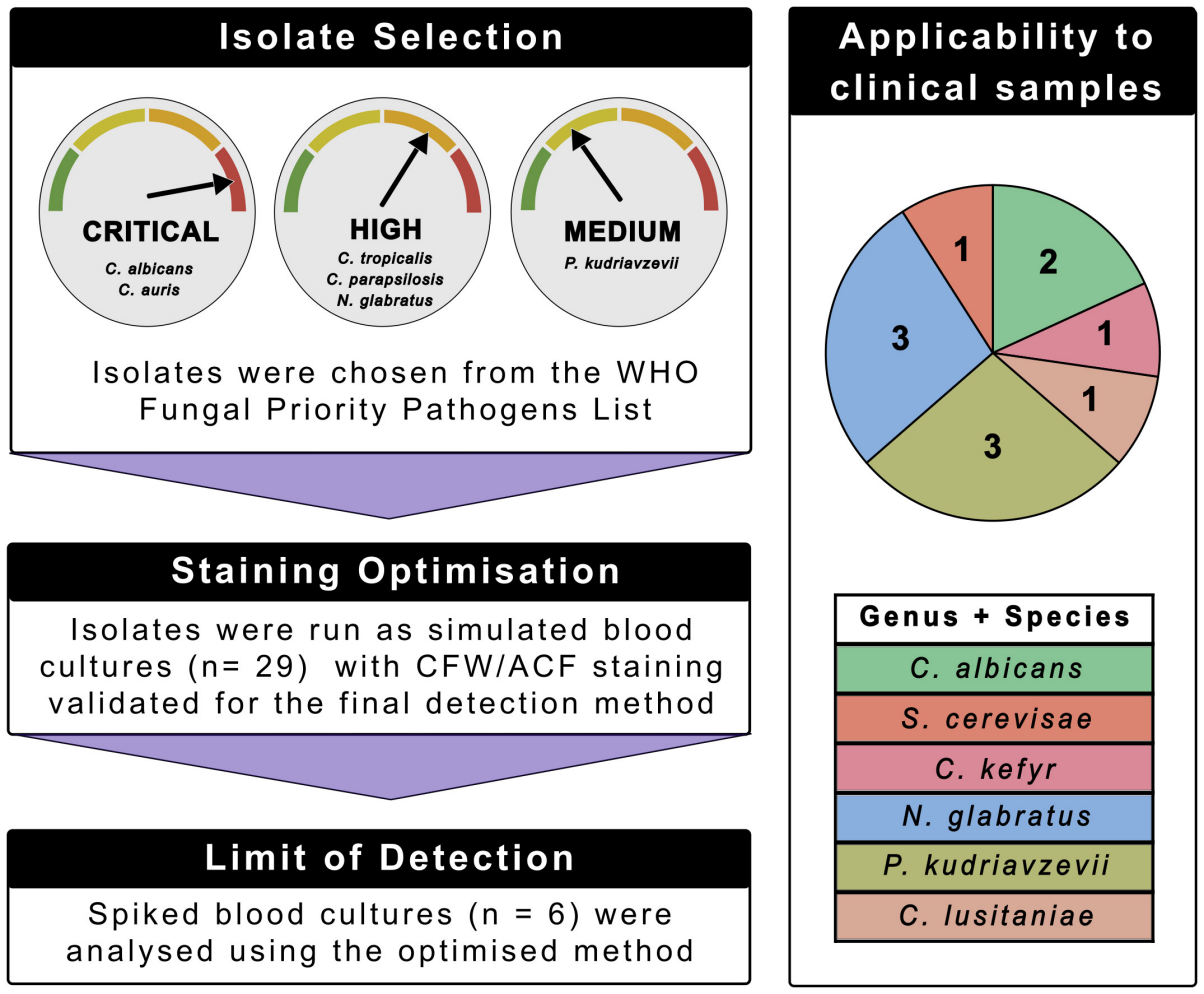
Diagram of study design and methods. A pool of representative yeast species were chosen from the WHO FPPL and used for marker selection. “Critical” level isolates included *C. albicans* (*n* = 5) and *C. auris* (*n* = 3). “High” level isolates included *C. tropicalis* (*n* = 6), *C. parapsilosis* (*n* = 5), and *N. glabratus* (*n* = 5) while *P. kudriavzevii* isolates (*n* = 5) were selected from the “medium” priority group. Isolates were used in spiked blood culture experiments to assess the performance of all fluorescent makers. The optimal staining combination (CFW and ACF) was then applied to both clinical blood cultures and the limit of detection study. Clinical blood cultures were processed if they flagged positive on the BD Bactec^TM^ FX with yeast or “yeast- like” cells visible on a Gram stain. The numbers of samples analyzed and isolate identification is shown in the pie chart and table, respectively.

To develop a rapid flow cytometric assay for yeast cell detection in blood cultures, a pool of clinically derived yeast isolates were used for spiked blood culture experiments (*n*= 29) (Figure 1). Yeast species were selected from the WHO FPPL (WHO, 2022), with the inclusion of multiple isolates responsible for clinical episodes of candidemia. We first investigated the performance and specificity of three established yeast cell stains: CFW, ConA, and ACF for the detection of yeast cells in blood cultures. The optimized stain selection (CFW and ACF) was then applied to spiked blood cultures to ensure all species would be detectable by flow cytometry. Spiked bacterial cultures (five control isolates) were also processed using the optimized method to assess for potential non-specific staining of the markers to bacterial cells. The method was then applied to limit of detection (LOD) experiments in spiked blood cultures (*n* = 6) and the clinical utility of the assay was evaluated using leftover, deidentified “yeast-positive” blood cultures submitted to the local pathology service (*n* = 11).

### 2.2 Microbial strains, fluorescent markers, and reagents

#### 2.2.1 Yeast isolates

Clinical isolates were identified using matrix assisted laser desorption ionization-time of flight mass spectrometry (MALDI-TOF MS - Bruker, Billerica, MA, United States) and were provided by PathWest Laboratory Medicine WA (Fiona Stanley Hospital and QEII Medical Centre, Perth, Australia). The following clinical yeast isolates were used in the study: *C. albicans* (*n* = 5), *C. parapsilosis* (*n* = 5), *N. glabratus* (*n* = 5), *C. tropicalis* (*n* = 6), *P. kudriavzevii* (*n* = 5), and *C. auris* (*n* = 3) (Figure 1). For the bacterial blood culture spike experiments, ATCC 10231 (*C. albicans*) and ATCC 15545 (*N. glabratus*) were used as positive controls. All yeast isolates were cultured on Sabouraud agar plates (SAB - PathWest Media, Perth, Australia). For long term storage, *Candida* isolates were inoculated in Yeast Extract-Peptone-Dextrose (YPD) prepared according to manufacturer's instructions (Scientifix, VIC, Australia) with 20% glycerol in cryopreservation tubes and stored at −80 °C.

#### 2.2.2 Bacterial isolates

The following bacterial isolates were used to assess staining specificity: ATCC 25922 (*Escherichia coli*), ATCC 700603 (*Klebsiella pneumoniae*), ATCC 27853 (*Pseudomonas aeruginosa*), ATCC 12228 (*Staphylococcus epidermidis*), and ATCC 25923 (*S. aureus*). Blood agar (BA) plates containing sheep blood (5% v/v) (PathWest Media, Perth, Western Australia) were used for culturing bacterial isolates. For long term storage, colonies were emulsified in Brain Heart Infusion Broth (BHI) with 20% glycerol (PathWest Media, Perth, Australia) in cryopreservation tubes and stored at −80 °C.

#### 2.2.3 Fluorescent markers

##### 2.2.3.1 Yeast marker optimization

Staining concentrations for CFW, ConA, and ACF were first investigated using two clinical yeast isolates (*C. albicans* and *N. glabratus*). Isolates were cultured on SAB plates for 48 h at 35 °C, colonies emulsified into 1 mL sterile 1X phosphate buffered saline (PBS) (Gibco, Life Sciences, CA, United States) serially diluted and stained with CFW for 5 min. Yeast cells were enumerated using the Attune Cytpix^TM^ (Thermo Fisher Scientific, Waltham, MA, United States) and standardized to approximately 2 × 10^6^ cells/mL in PBS. A 96 well plate (Greiner Bio-One, Kremsmünster, Austria) was prepared with the following concentrations (μg/mL) of yeast stains in PBS; CFW: 0.625, 1.25, 2.5, 5, and 10; ACF: 0.5–0.625, 1–1.25, 2–2.5, 4–5 and 8–10; ConA: 6.25, 12.5, 25, 50, and 100. Standardized yeast suspensions were added to the 96 well plate (excluding negative control wells) and diluted 1:10. Unstained controls (0 μg/mL of stain) were prepared in parallel for each isolate with negative controls (PBS with highest concentration of tested yeast stain). CFW and ACF samples were stained for 30 min at room temperature and ConA samples were stained for 30 min at 35 °C. Samples and controls were run in technical triplicate on the flow cytometer. The most cost-effective concentration with the highest signal to noise ratio was selected (Supplementary Figure 1) for each stain. After optimization, the final concentration of CFW—with a fixed ratio of Evan's Blue – (EB) was 5 μg/mL and 2.5 μg/mL, respectively (Sigma Aldrich, SL, United States). CF^®^488A Concanavalin A (ConA) (Biotium, Fremont, CA, United states) was used at a final staining concentration of 50 μg/mL and *Candida albicans* polyclonal antibody fluorescein isothiocyanate (Anti-*Candida* FITC - PA173154) (Thermo Fisher Scientific, Waltham, MA, United States) was used at a concentration of 4-5 μg/mL (1: 1,000 dilution).

##### 2.2.3.2 Human cell markers

Human cell markers, CD45 Monoclonal Antibody, Alexa Fluor^TM^700, (2D1) (Thermo Fisher Scientific) and CD235a (Glycophorin A) Monoclonal Antibody, APC-eFluor^TM^780, (HIR2, GA-R2) (Thermo Fisher Scientific) were used at a final concentration of 0.03 μg/mL.

##### 2.2.3.3 Bacterial cell markers

For bacterial staining, SYTO^TM^9 (Thermo Fisher Scientific) was used at a previously optimized final concentration of 5 μM (Mulroney et al., 2017).

##### 2.2.3.4 Staining times and conditions

Unless otherwise stated, pure yeast cultures were stained with CFW at room temperature in the dark for 5 min. Blood culture samples (post processing) were stained either with CFW and ACF (at room temperature) or CFW and ConA (at 35 °C) for 30 min in the dark prior to flow cytometric analysis. Samples with human cell markers (CD45 and CD235a) were stained on ice for 30 min.

#### 2.2.4 Ammonium chloride lysis buffer

Ammonium chloride (Sigma Aldrich, MO, United States), potassium bicarbonate (Sigma Aldrich) and EDTA (Sigma Aldrich) were added to 800 mL of MilliQ water for final concentrations of 0.15 M, 0.01 M, and 0.1 mM, respectively. The pH was adjusted to 7.2 and additional MilliQ water was added for a final volume of 1,000 mL. Aliquots were filtered (Millipore PVDF 0.2 μm syringe filter, Merck, Darmstadt, Germany) prior to use.

### 2.3 Preparation of spiked yeast blood cultures

Spiked blood cultures were prepared by supplementing commercial blood culture (BC) bottles (BD Bactec^TM^ Plus aerobic medium, Franklin Lakes, NJ, United States) with 10 mL of donor blood from healthy volunteers (University of Western Australia Human Research Ethics Committee Approval RA/4/20/5074 and 2024/ET000706) and a standardized inoculum of yeast test isolates (*n* = 29).

Yeast isolates were streaked onto SAB plates and incubated for 48 h at 35 °C. Blood culture inocula were prepared by emulsifying single colonies from SAB plates into 1 mL PBS. To enumerate cells, aliquots were serially diluted in 0.1 μm filtered Hank's Balanced Salt Solution (HBSS) with phenol red—(PathWest Media, Perth, Australia) and stained with CFW as described earlier. Yeast cell suspensions were adjusted in PBS and spiked into BC bottles supplemented with donor blood to yield a final concentration of 1 × 10^5^ cells/mL. To confirm yeast cell concentrations, spiked blood culture aliquots were removed, serially diluted in PBS and 100 μL volumes plated in triplicate onto SAB plates. Plates were incubated for 24–48 h at 35 °C and colonies counted. Spiked bottles were loaded into the BD Bactec^TM^ FX system (bioMerieux, Marcy-l'Étoile, France) automated incubator and once positive for growth (as per the manufacturer's instructions) were processed prior to staining. The ATCC yeast controls were prepared in the same manner, however bottles were not incubated in the BD Bactec^TM^ FX system but processed after 24 h of incubation.

### 2.4 Preparation of spiked bacterial blood cultures

Spiked bacterial blood cultures were prepared in the same manner as the yeast blood cultures except for the following changes listed: bacterial isolates were streaked onto blood agar and incubated for 24 h at 35 °C. After incubation, colonies were emulsified into PBS, samples were serially diluted and stained with SYTO^TM^ 9 to obtain cell counts. Spiked bottles were incubated for 24 h at 35 °C and then processed and stained.

### 2.5 Preparation of donor blood for human cell marker staining

#### 2.5.1 CD235a staining

Whole blood from a heathy donor was stained with CD235a and further diluted in PBS (with 1% FBS, Moregate Biotech, QLD, Australia) in separate tubes. Yeast and human cell markers were added and further incubated for 30 min at the temperatures described previously. Samples were centrifuged at 400 × g for 5 min and washed in 1 mL PBS (with 1% FBS). An unstained sample was prepared in parallel. Samples were re-suspended in 1 mL of PBS and analyzed on the Attune^TM^ NxT (Thermo Fisher Scientific).

#### 2.5.2 CD45 staining

To lyse erythrocytes, 1 mL of donor blood was mixed with 9 mL of ammonium chloride buffer. The sample was incubated on ice for 5 min then centrifuged at 400 × g for 5 min. The pellet was washed with PBS (with 1% FBS) and resuspended in 1 mL PBS. After incubation at 37 °C for 15 min, the sample was further diluted in additional PBS (with 1% FBS). The sample was split into separate tubes and stained with CD45 for 30 min. Yeast cell markers were added to the appropriate tubes, samples were stained and then run through the flow cytometer.

### 2.6 Final blood culture processing and staining method

**Figure 2:**
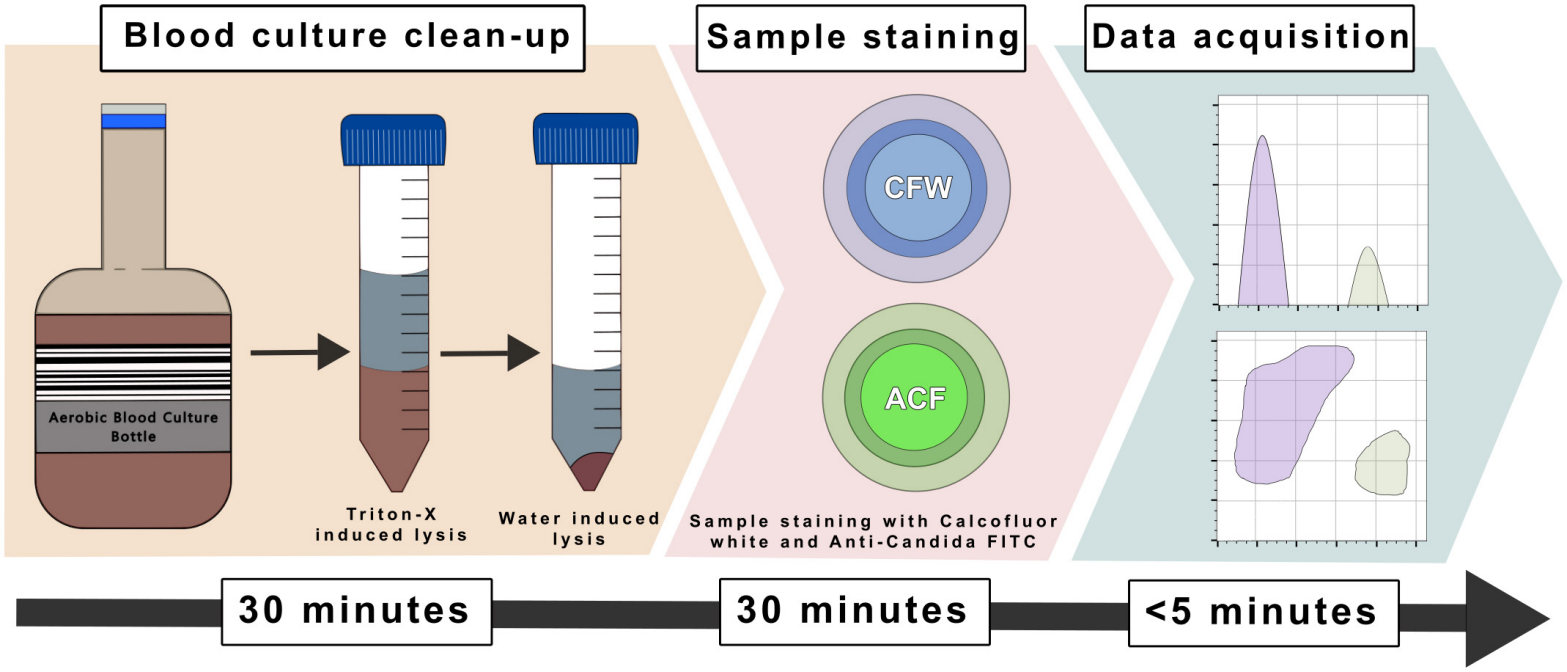
Diagrammatic illustration of final method for rapid yeast detection from blood cultures using flow cytometry. The time taken to perform each step is shown in the box at the bottom of the figure. For the blood culture clean-up, an aliquot of sample is removed from a blood culture bottle and incubated with Triton-X to lyse human cell components. Cells are pelleted, the waste removed and then further lysis is performed using sterile water. Re-suspended cells are then diluted and stained with Calcofluor white and Anti-*Candida* FITC. After staining, samples are run through the flow cytometer for the detection of yeast cells.

For the final method (Figure 2), spiked blood culture bottles were shaken and 5 mL of medium removed to a 15 mL centrifuge tube (Corning, New York). A 10 μL aliquot of sample was removed, plated on BA and incubated for 24–48 h to check for purity. Meanwhile, 5 mLs of 0.5% Triton^TM^ X-100 solution (Sigma-Aldrich, SL, United States), prepared in sterile water were added to the tube, thoroughly vortexed and incubated at 35 °C for 10 min. Tubes were spun at 1,600 × g for 5 min and the supernatant discarded. The remaining pellet was re-suspended in 5 mL sterile water and incubated at 35 °C for 10 min to induce hypotonic lysis of human cells. Tubes were then spun again and the supernatant discarded. The pellet was re-suspended in 1 mL HBSS or PBS with serial dilutions performed in HBSS/PBS prior to staining and acquisition by flow cytometry with results available in just over 1 h. If visible aggregates of hyphae were evident in the re-suspension, samples were passed through a 70 μm cell strainer (Greiner Bio-One, Kremsmunster, Austria) prior to staining to prevent machine clogging.

### 2.7 Spiked bacterial blood culture processing

Spiked bacterial blood culture bottles were processed, serially diluted into separate tubes and stained with SYTO^TM^9 as previously described prior to data acquisition. The same serial dilution without SYTO^TM^9, was then stained with CFW and ACF as described earlier. After staining, 250 μL of the suspension was transferred to a 96 well plate (Greiner Bio-One) in triplicate and run on the Attune^TM^ CytPix^TM^ with the Autosampler (Thermo Fisher Scientific). Unstained samples were run first, followed by stained samples then “blank” wells with PBS to minimize event carry over between samples. The method was repeated for the negative control (un-spiked human blood) and the yeast positive controls (ATCC 10231 and ATCC 15545).

### 2.8 Gating strategy validation using the Attune^TM^ CytPix^TM^

A clinical *C. albicans* isolate was spiked into 2 mL of donor blood for a standardized concentration of 3 × 10^6^ cells/mL, mixed and cleaned-up as described previously. The processed sample was resuspended in 1 mL of PBS and diluted 1:10 in HBSS then stained with CFW and ACF. Data was acquired using the imaging flow cytometer. To determine the potential misclassification of cells, 600 events were randomly sampled from a total of 1,796 events captured in the YER by the Cytpix^TM^ (Thermo Fisher Scientific). Images of these events were visually inspected using Image J (v 1.54 p) and cells defined as yeast based on morphological characteristics including: size, shape, the presence of distinct cell walls and cells with a high refractivity (higher contrast to background).

### 2.9 Confocal microscopy

To visualize the staining specificity of the yeast cell markers a clinical *C. albicans* isolate was spiked into a blood culture bottle, incubated for 48 h, then processed and stained as described earlier. After staining, the samples were diluted 1:10 in PBS and spun at 12, 000 × g for 5 min and the supernatant discarded. Cells were fixed in 4% formaldehyde (Pierce^TM^ 16% Formaldehyde (w/v), Methanol-free, ThermoFisher Scientific) for 20 min at room temperature, pelleted and then re-suspended in 50 uL PBS. Ten microliters of sample was added to Poly-L-Lysine coated slides (ProSciTech, Kirwan, QLD, Australia) and air dried in a biosafety cabinet. One drop of glycerol mounting medium was added (refractive index of 1.472), (ProSciTech) and covered with a coverslip (0.17 mm thickness) (ProSciTech). The sides were sealed with clear nail polish and air dried in the dark. Imaging was performed using the Nikon A1Si confocal microscope (Tokyo, Japan) (with a 20 × , 0.75-numerical-aperture dry lens, 3 × digital zoom) and the following lasers: 405 nm, 495 nm, and 640 nm. Sequential scanning was used, and images were collected using the NIS elements software (Version no: 5.02.03).

### 2.10 Limit of detection in blood cultures

After fluorescent marker selection, the optimized method was applied to spiked blood cultures to determine the limit of detection (LOD) using flow cytometry and plate counts. Yeast isolates were grown on SAB as described earlier (*n* = 6, one isolate of each species) with single colonies emulsified into 10 mL YPD and grown for an additional 24–48 h at 35 °C. Overnight suspensions were enumerated using CFW as previously described and diluted to 3 × 10^6^ cells/mL in PBS. Standardized inocula were prepared by serially diluting this suspension to a final concentration of 3 × 10^1^ cells/mL in separate tubes. Multiple aliquots of 2 mL were sampled from a BC bottle supplemented with donor blood. One milliliter of standardized inocula or PBS (negative control) were added to the 2 mL aliquots. The blood culture clean-up process was performed immediately using Triton^TM^ X-100 and water as previously described, with a final resuspension in 1 ml HBSS. For flow cytometric processing, samples were diluted 1:10 in HBSS and stained as described earlier. Unstained samples were run in parallel on the Attune NxT (Thermo Fisher Scientific, Waltham, MA, United states) After staining, 250 μL of each sample was aliquoted into a 96 well plate (Thermo Fisher Scientific, Waltham, MA, United States) in triplicate and data acquired using the Attune^TM^ NxT autosampler. For plate counts, serial dilutions of the re-suspended sample were prepared in PBS and 100 μL plated onto SAB plates in triplicate. Plates were incubated at 35 °C for 24–48 h and colonies counted.

### 2.11 Application to clinical blood cultures

We applied our optimized assay to deidentified spent blood culture clinical samples provided by a local pathology service, PathWest Laboratory Medicine, QEII Medical Centre, Perth, Australia (Figure 1). The activity was exempt from ethical approval according to guidance in the National Statement on the Ethical Conduct of Research in Humans (National Statement on Ethical Conduct in Human Research 2025 | NHMRC, 2025). Aliquots of 5 mL of positive blood culture samples were cleaned and processed on the Attune^TM^ NxT as described earlier if they: flagged positive for growth on the BD Bactec^TM^ FX with yeast or “yeast-like” cells visible on a Gram-stain. Cleaned samples were dual stained with CFW and ACF, serially diluted in PBS and run on the flow cytometer. Plate counts were performed in parallel as described earlier. Species were identified by the pathology lab using the MALDI-TOF MS. Clinical samples analyzed included the following species: BC01 (*S. cerevisae*), BC02 (*P. kudriavzevii*), BC03 (*P. kudriavzevii*), BC04 (*N. glabratus*), BC05 (*C. kefyr*), BC06 (*C. albicans*), BC07 (*N. glabratus*), BC08 (*N. glabratus*), BC09 (*Clavispora lusitaniae)*, BC10 (*P. kudriavzevii*), and BC11 (*C. albicans*).

### 2.12 Acoustic flow cytometer operation and settings

#### 2.12.1 Attune^TM^ NxT and Attune^TM^ Cytpix

Both flow cytometers were calibrated prior to each run according to the manufacturer's instructions (ThermoFisher Scientific) For yeast markers, flow cytometer settings were: Forward Scatter (FSC) voltage 100, FSC threshold 0.8 × 1,000 AND, Side Scatter (SSC) voltage 240, SSC threshold 0.7 × 1,000 AND, violet laser 1 (VL1 – 440/50 nm) (CFW) 185, blue laser 1 (BL1 – 530/30 nm) (ConA/Anti-Candida FITC) 310, and red laser 1 (RL1 – 670/14 nm) (EB) 300. The same settings were used for the human cell markers with the additional channels used: (CD45), 330, red laser 2 (RL2 - 720/30 nm) and (CD235a), 350, red laser 3 (RL3 – 780/60 nm). For bacterial spike experiments, the following settings were used to detect bacteria on scale: FSC voltage 340, FSC threshold 0.3 × 1,000 AND, SSC voltage 340, SSC threshold 0.2 × 1,000 AND, BL1 voltage 260 (SYTO^TM^9), BL1 threshold 0.1 × 1,000 AND, BL2 voltage 200.

**Figure 3:**
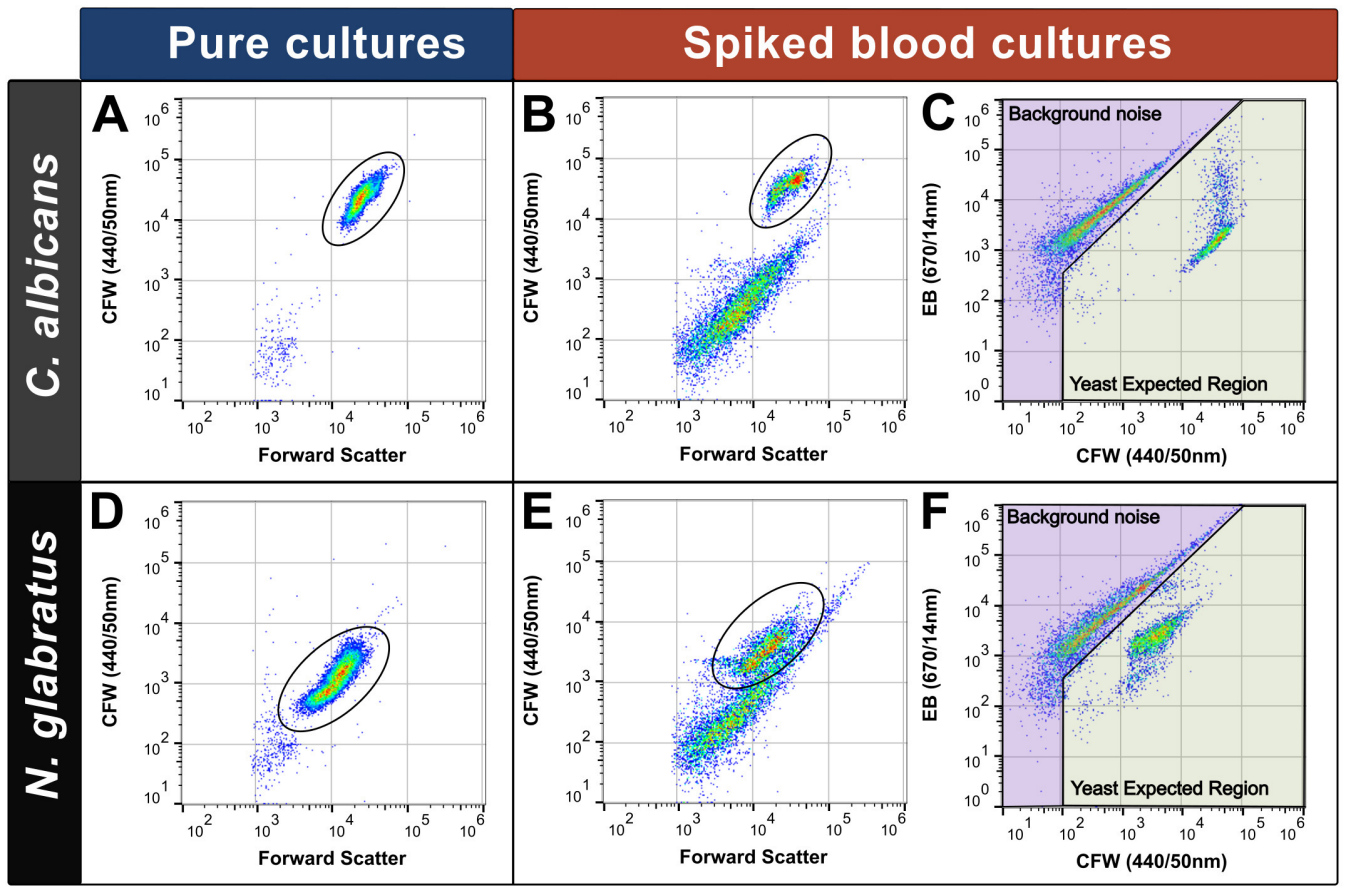
Spiked blood cultures require a different gating strategy to pure cultures for optimal separation of yeast and human cells. Minimal background noise was observed in pure cultures **(A, D)** compared to spiked blood cultures **(B, E)** (yeast cells gated in black oval). Some species stained lower in Calcofluor White (CFW) with some yeast cell events obscured by background noise (*N. glabratus* – E). Separation of yeast cells from background noise was achieved by plotting data on a biaxial plot (CFW and Evan's Blue fluorescence) **(C, F)** with yeast cells sitting in the “Yeast Expected Region.”

**Figure 4:**
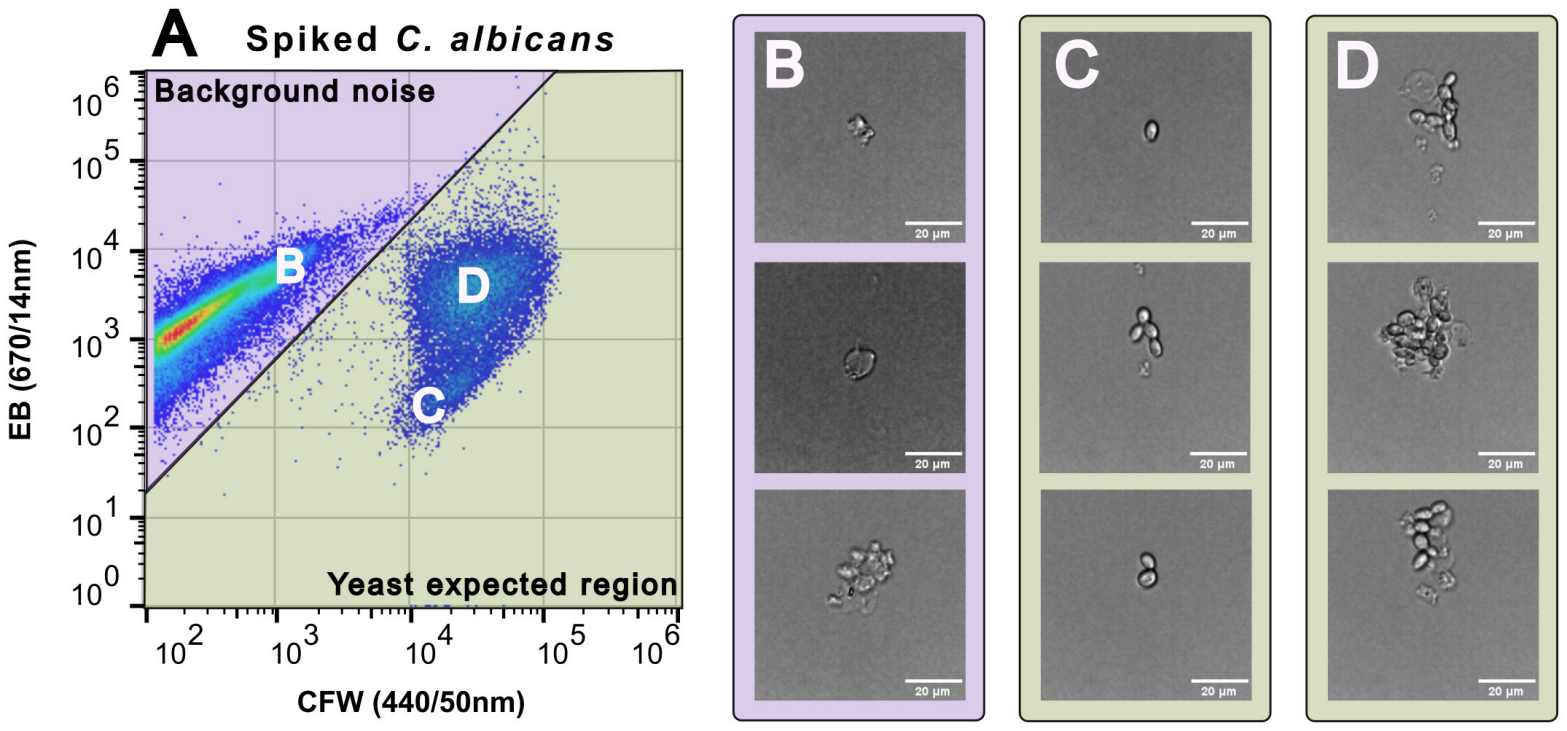
Gating outcomes using the Attune Cytpix^TM^ with microscopy images of different cell populations. **(A)** Pseudo-color plot of spiked *C. albicans* blood culture (3 × 10^6^ cells/mL) after clean-up and staining with CFW and ACF. Cell populations were separated on the biaxial plot into a purple shaded background noise area with corresponding representative Cytpix images in panel **(B)** (human cells and debris). Cell populations in the yeast expected region are shown in panel **(C)**, and with higher EB fluorescence in panel **(D)**. Large yeast cell aggregates adhering to human cells and debris were observed in panel **(D)**. The scale bar of 20 μm is shown on all Cytpix^TM^ images ( × 20 magnification).

Unless otherwise stated a flow rate of 100 μL/min was used for experiments with an acquisition volume of 250 μL. Acquisition was halted after 10, 000 events or 50 μL were collected. For bacterial spike experiments a flow rate of 200 μL/min was used and acquisition halted after 60 μL were collected. For the Cytpix experiment, acquisition was halted after 100 μL of sample were collected. For LOD experiments, the acquisition was halted after 10, 000 events collected in the Yeast Expected Region (YER) (examples shown in Figures 3, 4) or 150 μL of sample were collected. Each sample was acquired in technical triplicate.

### 2.13 Flow cytometry controls

In parallel to stained samples, an unstained control was also analyzed to check for any auto fluorescent cell populations. Background controls (HBSS or PBS stained with fluorescent markers) were also included for each run to analyze the background noise of the diluent. For the spiked blood cultures and LOD experiments, aliquots of un-spiked blood were processed and stained.

### 2.14 Bacterial cell gating

Bacteria stained with SYTO^TM^9 were gated according to our previously published method (Mulroney et al., 2017). Cell aggregates were removed using the same strategy except SSC-H and SSC-A were the chosen parameters and a 10% contour plot was not used for final gating.

### 2.15 Human cell gating

Files were concatenated (technical triplicates) and doublets removed using SSC-H/SSC-A. The CD235a+ and CD45+ populations were plotted on a histogram and the final gate drawn on the population using forward scatter and the channel of interest for each human marker. The gate was then applied to all other samples stained with the human cell marker and one other yeast cell marker (either CFW, ACF or ConA). The unstained control was used to exclude autofluorescence in each channel and after application of the CD235a or CD45 gate, any cells with a positive signal in the yeast marker channels were gated.

### 2.16 Data analysis and statistics

Flow cytometric data were exported and analyzed using FlowJo v10.10.0 (BD Life Science, Ashland, USA). Images from the Cytpix were exported as TIF files and uploaded into Image J (v 1.54p). The pixel width and height of the image were changed to 0.3 μm and a 20 μm scale bar was added with images exported again as TIF files. Statistical analysis was performed using GraphPad Prism v10.2.3 (GraphPad Software, San Diego, USA). Normality tests (Shapiro-Wilk) were performed using a 0.05 significance level. For bacterial spike data, differences in the percentage of cells stained (Gram-negative bacteria and negative controls) were accessed using a Kruskal-Wallis non-parametric one-way ANOVA, with Dunn's multiple comparisons.

The LOD was calculated from the equation provided by Illingworth et al. (2018):


$$\begin{array}{l} LOD=Mean\;of\;blank+3\;\times SD \end{array}$$

with the mean of blank referring to the concentration of cells in the un-spiked blood controls after final gating. For normalization, cell concentrations were log_10_ transformed prior to final calculations. The mean recovery efficiency was calculated from plate count data following log_10_ transformation.

## 3 Results

### 3.1 ConA exhibits the highest level of non-specific binding to human cells compared to the other markers

**Figure 5:**
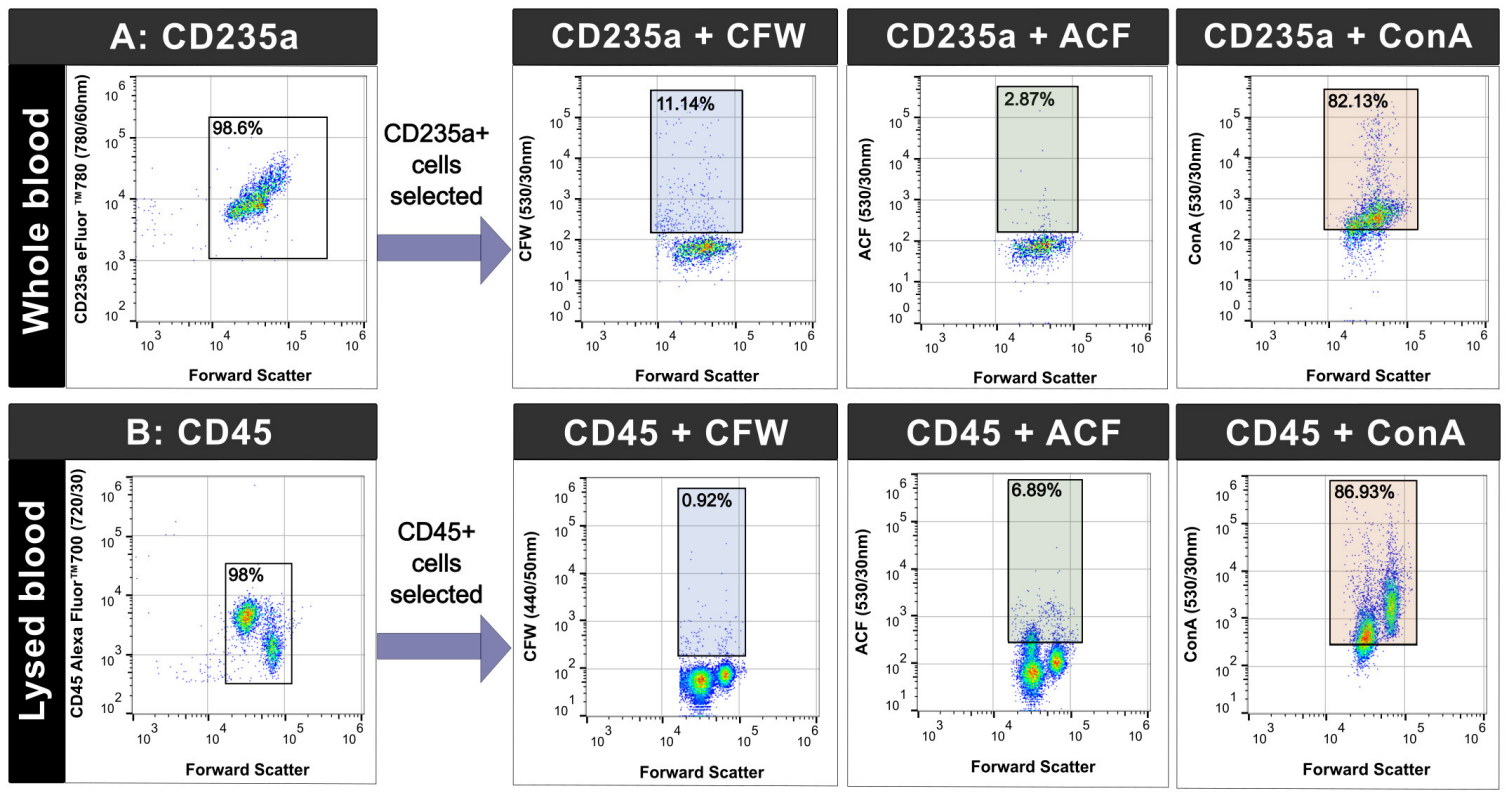
Concanavalin A (ConA) stains a higher percentage of CD235a and CD45 positive cells compared to Calcofluor White (CFW) and Anti-*Candida* FITC (ACF) (concatenated files shown). Whole donor blood was stained with CD235a **(A)** and combined with CFW, ACF, and ConA in separate aliquots. Lysed blood was stained with CD45 **(B)** and combined with CFW, ACF, and ConA were in separate aliquots. Unstained controls were used to set the gates for the yeast markers. Percentage of cells positive (mean value) for each marker is shown in the colored boxes.

We stained CD235a (Figure 5A) and CD45 (Figure 5B) positive cells in donor blood with combinations of different yeast markers. In whole blood (Figure 5A), the ACF stained sample had the lowest mean percentage of non-specific binding to CD235a+ cells (2.78%, SD = 0.99) while the ConA stained sample had the highest (82.13%, SD = 0.57). In the lysed blood sample (Figure 5B), CFW exhibited the lowest mean percentage of non-specific binding to CD45+ cells (0.92%, SD = 0.12) while ConA had the highest percentage (86.93%, SD = 0.25) out of the three markers.

**Figure 6:**
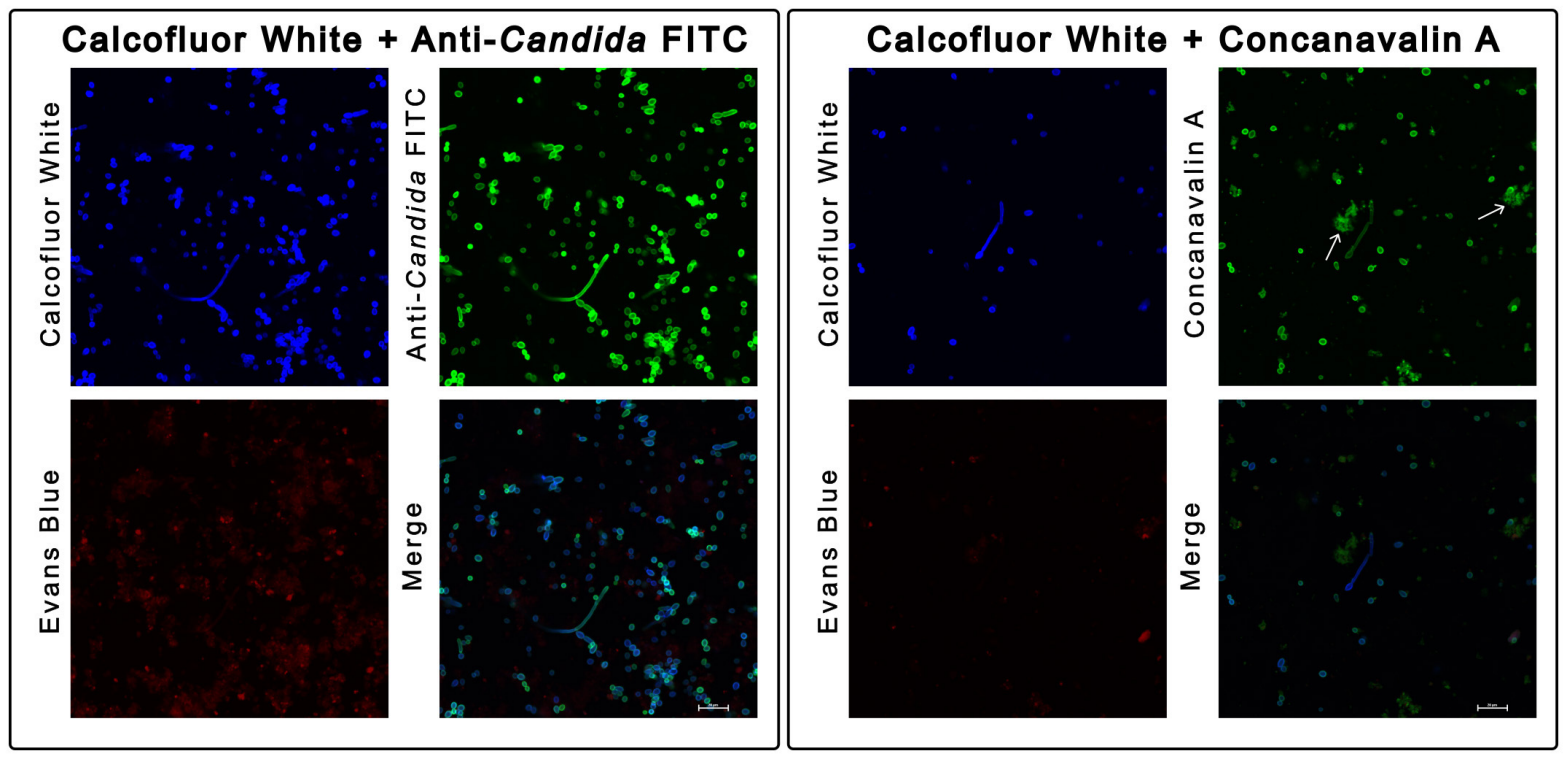
Concanvalin A (ConA) stains additional human cells compared to Calcofluor White (CFW) and Anti-*Candida* FITC (ACF). Confocal microscopy images of *C. albicans* stained and fixed from a spiked blood culture bottle (20 × magnification, 3 × digital zoom). The two panels display different staining combinations of two aliquots of the same sample, with individual images showing specific optical filters. First panel, left to right: CFW staining, ACF, Evan's Blue (EB), and merged image. Second panel, left to right: CFW staining, ConA, EB, and merged image. White arrows in the second panel (ConA staining) point to fluorescently labeled non-yeast cell fragments. Scale bar for all images is 20 μM.

The non-specific staining dynamics of ConA were also confirmed using confocal microscopy (Figure 6) in a spiked *C. albicans* blood culture sample. CFW and ACF were observed to specifically stain yeast cells, while non-yeast cell particles (shown using white arrows) displayed varying degrees of non-specific ConA fluorescence. After these observations, ConA was excluded from the final marker selection (to reduce potential false positives), and we proceeded to stain samples using CFW and ACF.

### 3.2 Gating strategy optimization and validation

In pure-culture samples stained with CFW, background noise was minimal (Figures 3A, D), and single stain gating was used to enumerate yeast cells. This method was highly accurate for the preparation of standardized yeast cell suspensions in donor blood across all species tested (Supplementary Figure 2) (Target count = 5.0 log_10_ CFU/mL, mean count obtained = 5.005 log_10_ CFU/mL, SD = 0.14). In spiked blood cultures the additional background noise overlaid yeast populations such as *N. glabratus* which displayed overall dim bi-modal CFW staining (low population: 42.8% gated cell events, log_10_ MFI −2.82; high population: 57.2% gated cell events, log_10_ MFI −3.230) compared to *C. albicans* (log_10_ MFI −4.35). Plotting data using CFW and EB fluorescence gave the greatest separation (Figures 3C, F) with yeast cells sitting in the “Yeast cell Expected Region” (YER) (highlighted in green). We used a manual gating strategy to define and separate the two populations (yeast cells from background noise). We validated assumptions about cell identities within gated regions using Cytpix^TM^ imaging flow cytometry (Figure 4). Events residing in the background noise region (purple) consisted of debris and human cells (Figure 4B) with yeast cells of various morphotypes residing in the YER (Figure 4C). Larger yeast and human cell aggregates were also observed in the YER (Figure 4D) with this subpopulation staining brighter in EB. Of the events falling within the YER, 94.67% were consistent with yeast morphology characteristics.

### 3.3 CFW binds more non-specifically to gram-positives compared to gram-negatives

**Figure 7:**
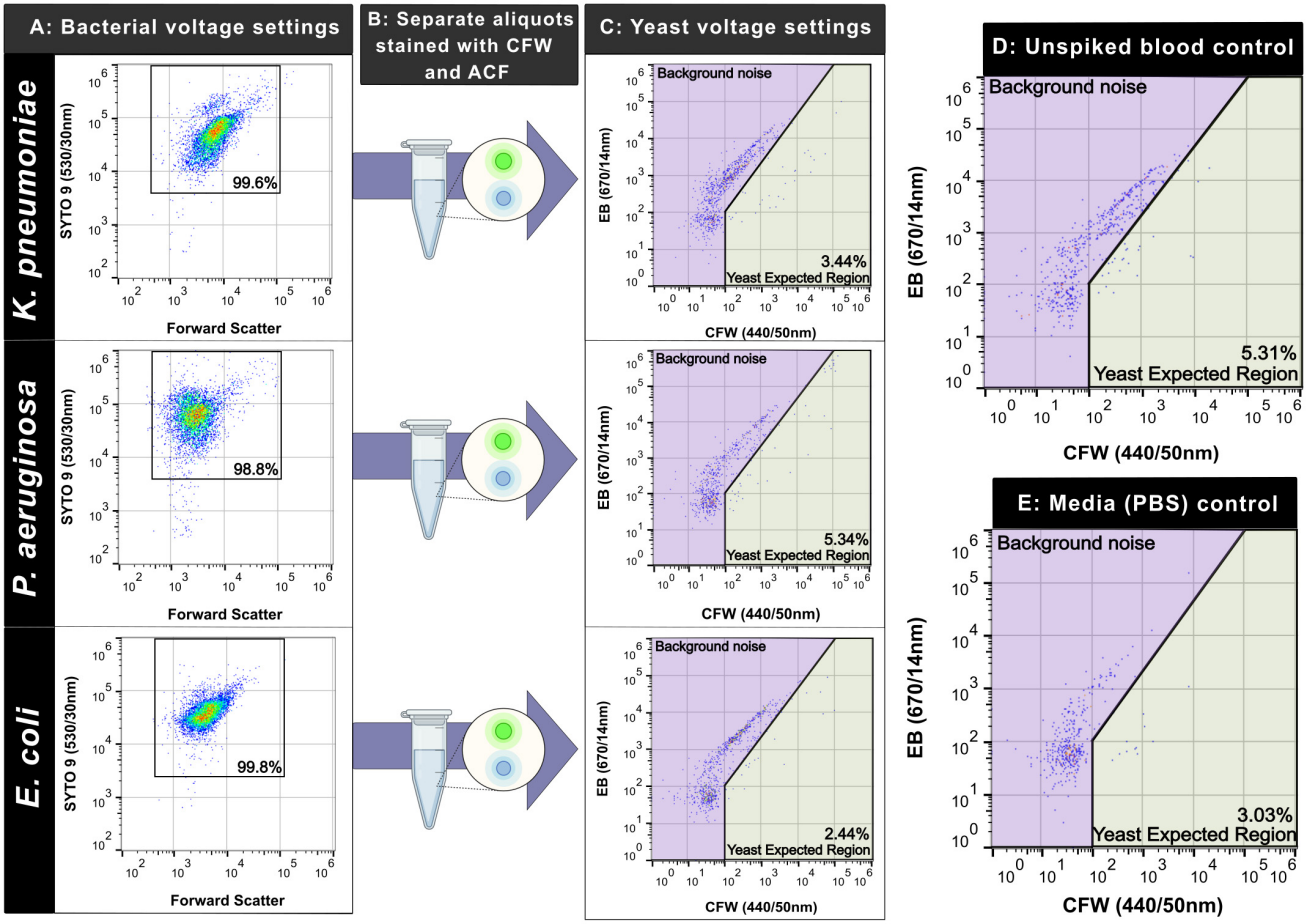
Calcofluor White (CFW) does not stain Gram-negative bacteria in spiked blood cultures. Plots and percentages from one experiment shown. **(A)** Pseudo-color plot of bacterial species stained with SYTO^TM^9 after blood culture processing. The percentage of gated cells (SYTO^TM^9 +ve) is shown in the black box. **(B)** Separate aliquots of the same sample were stained with CFW and ACF (no SYTO^TM^9 added). **(C)** Pseudo-color plots of CFW- and ACF-stained samples run at yeast-optimized voltage settings (CFW and EB fluorescence only shown). The percentage of events in the yeast expected region are shown in the green region. **(D, E)** Plots of controls run including unspiked blood and media - phosphate-buffered saline (PBS). Icons created in BioRender. Kopczyk, M. (2026) https://BioRender.com/vow2gqe.

**Figure 8:**
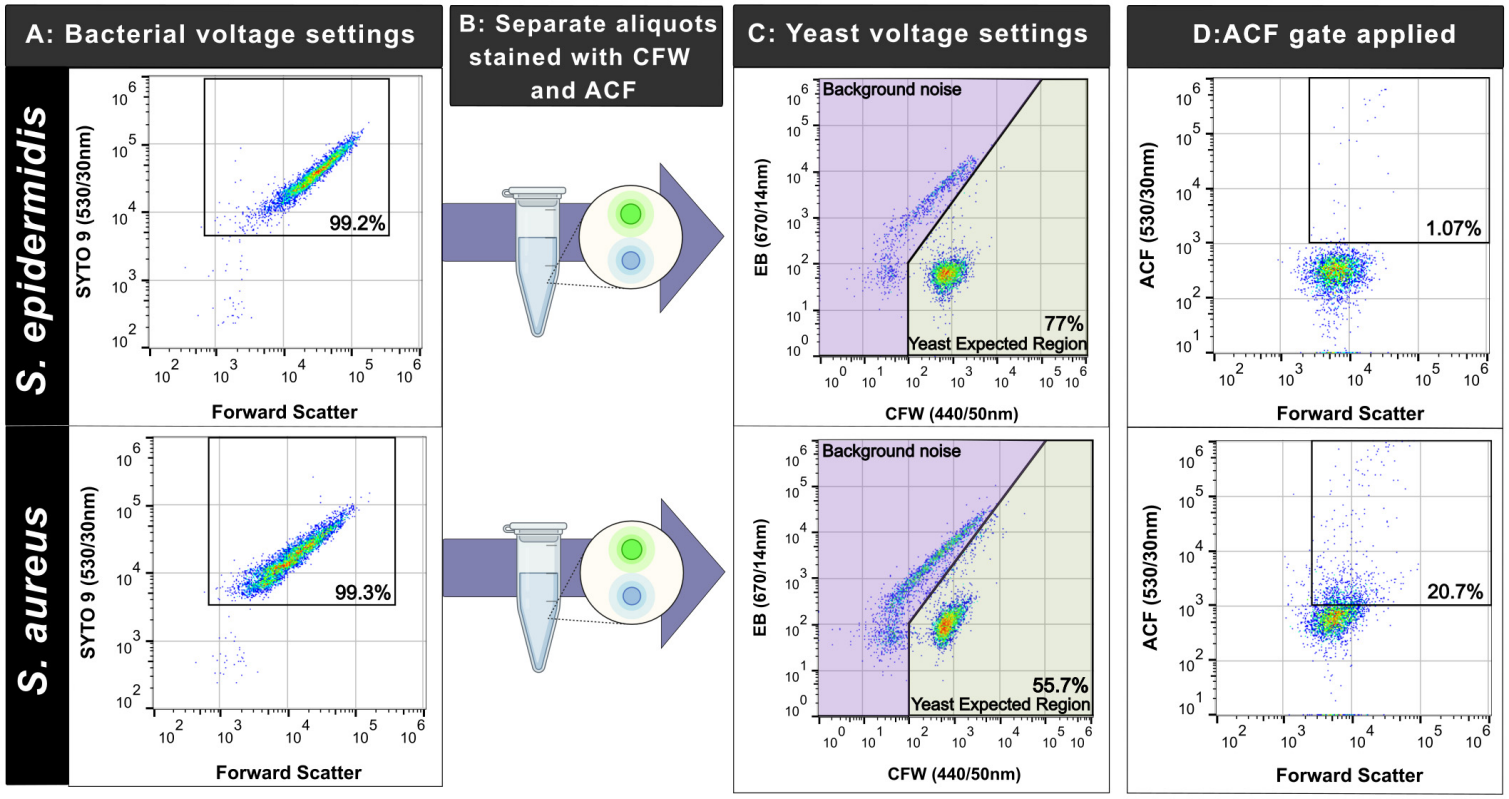
Calcofluor White (CFW) labels Gram-positive bacteria in spiked blood cultures necessitating the use of a second marker (Anti-*Candida* FITC) (ACF) to improve assay specificity. Plots and percentages from one experiment shown. **(A)** Pseudo-color plot of bacteria stained with SYTO^TM^9 post blood culture processing. The percentage of gated cells (SYTO^TM^9 +ve) is shown in the black box in the first panel. **(B)** Separate aliquots of the sample were then stained with CFW and Anti-Candida FITC (ACF) (no SYTO^TM^9 added). **(C)** Pseudo-color plots of CFW and ACF stained samples run at yeast-optimized voltage settings. Percentage of events in the yeast expected region are shown in the green region. **(D)** Application of the ACF gate to cells in the YER region. Percentage of positive events are shown in the black box. Icons created in BioRender. Kopczyk, M. (2026) https://BioRender.com/vow2gqe.

We assessed the specificity of our staining workflow against a variety of Gram-negative and positive bacteria. Spiked, simulated blood cultures containing bacteria were used as the comparator, enumerated with the optimized SYTO^TM^ 9-based method (Figures 7, 8). In separate aliquots stained with the yeast markers, we saw minimal non-specific staining in the Gram-negatives: with mean non-specific positive staining percentages of 2.44% (SD = 1.51%), 2.81% (SD = 1.49%), and 2.77% (SD = 0.73%) for *K. pneumoniae, P. aeruginosa*, and *E. coli* respectively. There was no statistically significant increase in non-specific detection compared to the negative controls-−3.39% (SD = 1.16%) for the blood-only control, and 2.67% (SD = 2.63%) for the PBS control (*p* = 0.31). Both *S. aureus* and *S. epidermidis* showed high non-specific staining with CFW alone-−71.62% (SD = 10.98%) and 68.41% (SD = 6.23%), respectively (Figure 8). Inclusion of an ACF gating step, requiring a “double positive” detection to be classified as a yeast cell, restored the assay specificity as mean event percentages dropped to 31.63% (SD = 8.64%) (*S. aureus*) and 1.98% (SD = 0.91%) (*S. epidermidis*). For the positive controls, mean event percentages after gating were 75.78% (SD = 9.89 %) for *C. albicans* and 94.69% (SD = 7.11%) for *N. glabratus*. The summary of staining patterns in the three markers tested is shown in Table 1.

**Table 1:** Summary of cell-types stained with Calcofluor White (CFW), Concanavalin A (ConA), and Anti-Candida FITC (ACF).

Fluorescent stains	Yeast	Gram-negative bacteria	Gram-positive bacteria	Human cells
CFW	+	-	+	-
ConA	+	NT	NT	+
ACF	+	-	-	-

Positive staining is highlighted in green (+); dim/no positive staining is highlighted in red (-), and cell populations that were not tested are shown in gray (NT).

### 3.4 Final gating strategy

**Figure 9:**
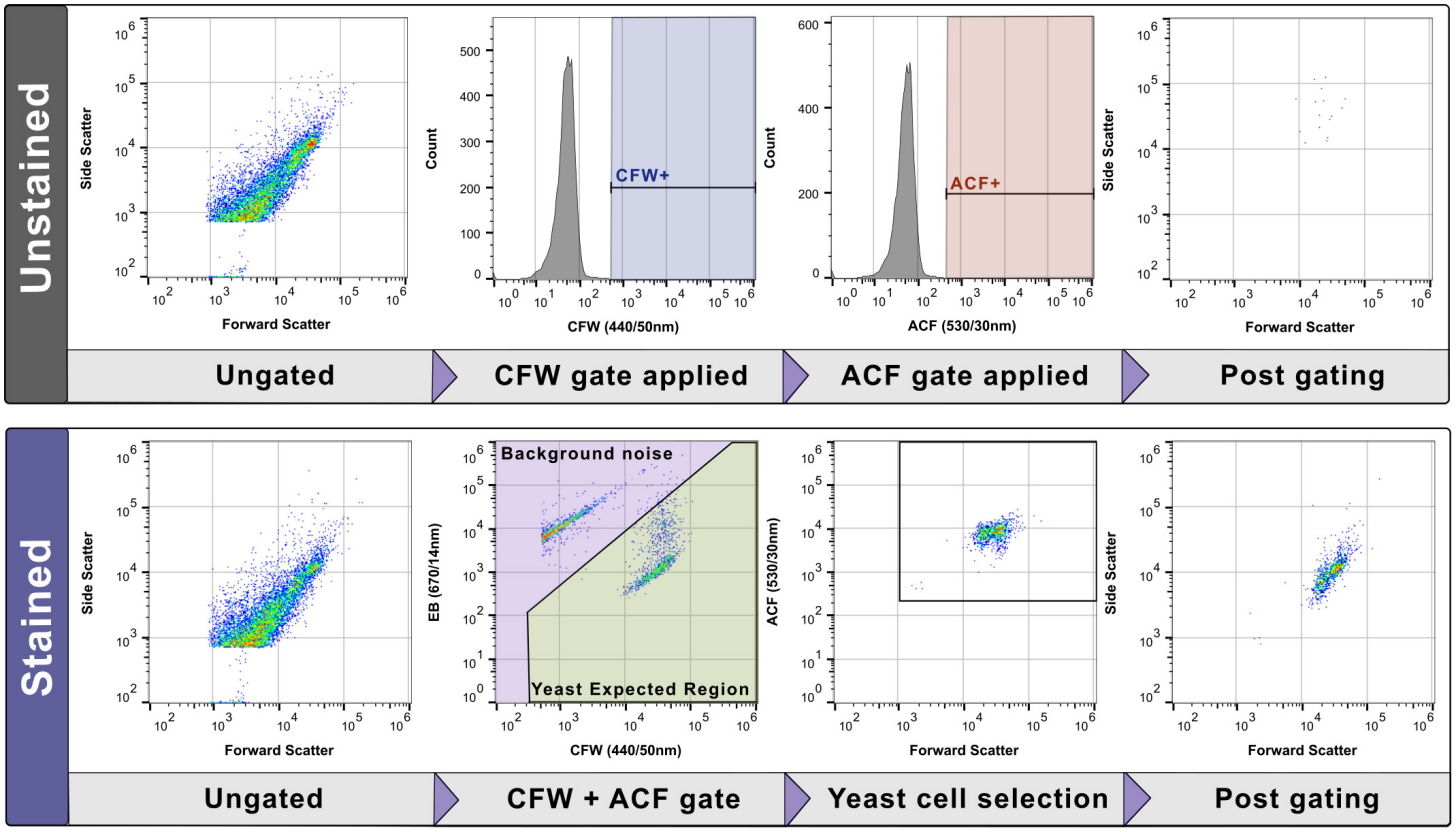
Final gating strategy for the detection of yeast in blood cultures (spiked *C. albicans* data shown). Using the unstained control for each sample, autofluorescence was excluded and the marker specific positive region set for both Calcofluor White (CFW) and Anti-*Candida* FITC (ACF). These gates were then applied to the stained sample with events selected in the Yeast Expected Region (YER) (green boxed area). The ACF gate is then applied to further improve the specificity of the gating strategy (exclude CFW non-specific staining to bacterial cells).

After investigating non-specific binding of the stains, our stain selection was finalized to CFW and ACF and our gating strategy designed to exclude non-yeast cell events (such as human cells, debris, and bacterial cells). The unstained control data was used to exclude auto fluorescent events and to define positive signals in both CFW and ACF (Figure 9). These gates are applied to the stained sample to exclude background noise, so that only cell events in the YER region were gated and selected. Finally, to exclude any bacteria that may have stained non-specifically with CFW, the ACF gate was applied to enable examination of various metrics such as counts of the yeast cell population.

### 3.5 Limit of detection in spiked blood cultures and applicability to clinical blood cultures

**Figure 10:**
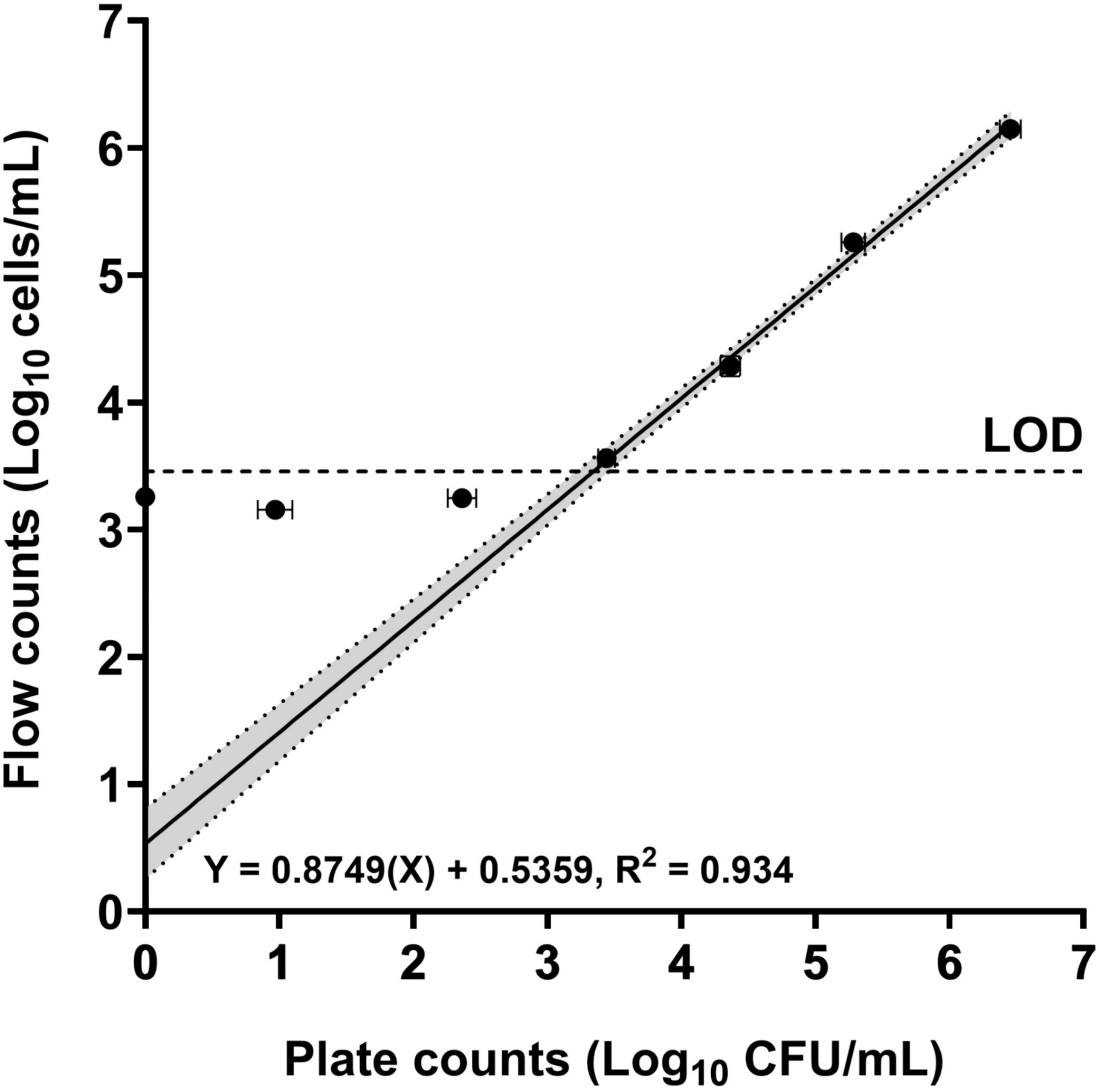
Limit of detection in spiked blood cultures (*n* = 6). Mean spiked yeast counts are shown (left to right): Negative control (0 cells/mL), 3 × 10^1^ cells/mL, 3 × 10^2^ cells/mL, 3 × 10^3^ cells/mL, 3 × 10^4^ cells/mL, 3 × 10^5^ cells/mL, and 3 × 10^6^ cells/mL. Mean counts are shown (log_10_ transformed) and error bars show SEM. The LOD is depicted as a dashed line (3.458). The line of regression is plotted on the countable range (3 × 10^3^ cells/mL to 3 × 10^6^ cells/mL), and the 95% CI is shaded gray.

**Figure 11:**
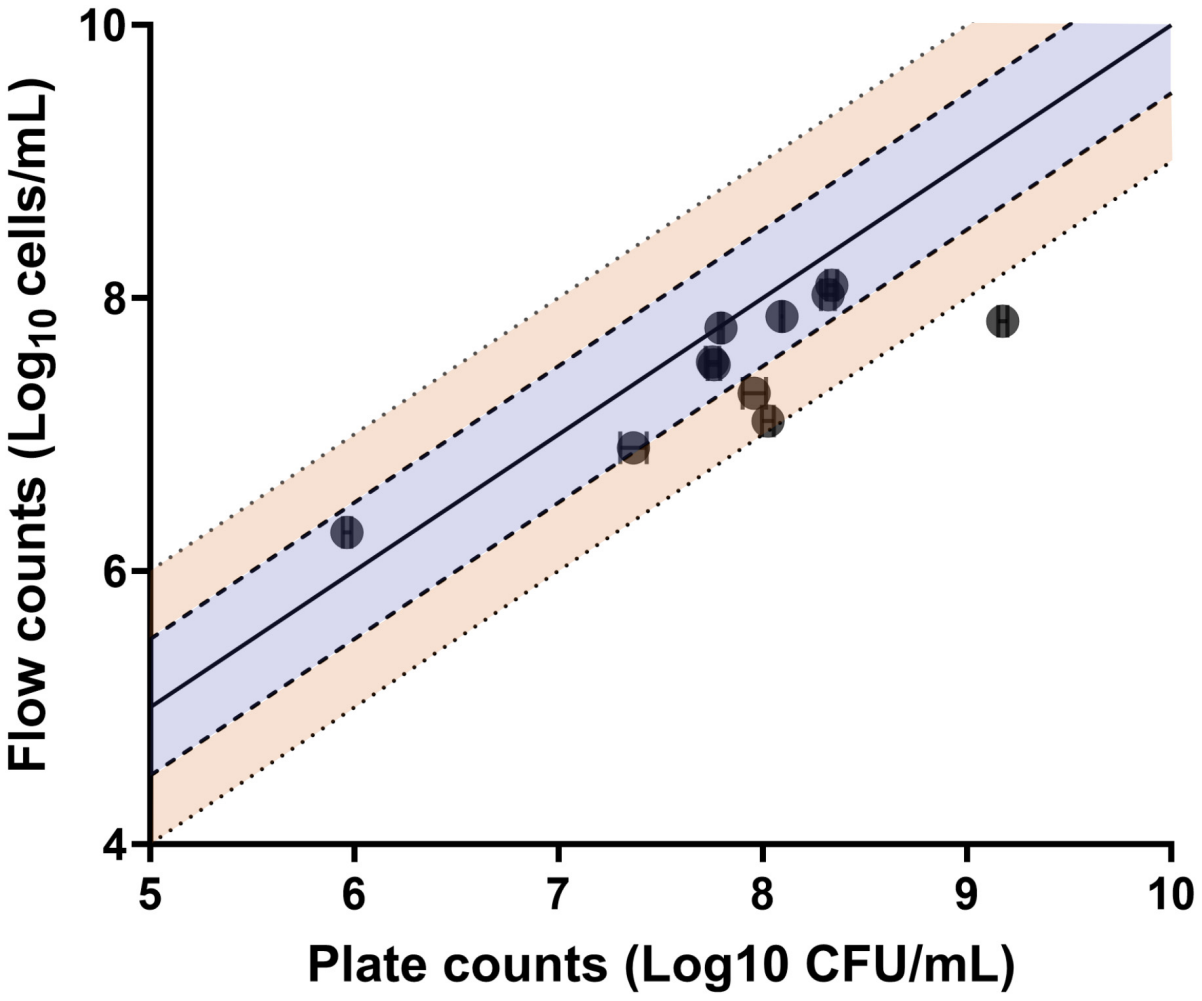
Flow cytometric and corresponding plate counts of clinical yeast positive blood cultures (*n* = 11). The mean count of each isolate is shown (log_10_ transformed and error bars depict SEM) with the line of identity (X = Y) as a solid line in the middle. The shaded orange section shows agreement within ±1log_10_ CFU/mL while the blue section shows agreement within ±0.5log_10_ CFU/mL to plate counts. BC09 (*C. lusitaniae)* is plotted outside the lines of agreement.

The mean limit of detection in spiked blood cultures using conventional flow cytometry was 2.871 × 10^3^ cells/mL (SEM = 1.050 × 10^1^ cells/mL) showing excellent correlation with growth-based methods (*R*^2^ = 0.934, Figure 10) and high technical reproducibility (mean replicate %CV = 6.458, SD = 2.25, Table 2). The mean cell recovery efficiency was 96.09% (SD = 17.66%) across the range of spiked concentrations tested after sample processing. Our method was successful in detecting and enumerating yeast cells in the blood cultures tested from a clinical laboratory. We could detect six different species including *Saccharomyces cerevisiae, C. kefyr*, and *C. lusitaniae*. The flow cytometric counts of 8 clinical samples tested were within ±0.5log_10_ of plated colony counts and 2 were within ±1log_10_ (Figure 11). The flow cytometric counts of one isolate (*C. lusitaniae)* were outside both ±0.5log_10_ and ±1log_10_ cells/mL to plate counts.

**Table 2:** Coefficient of variation percentage (CV%) in the limit of detection experiment in spiked blood cultures (flow cytometric and plate counts).

Spiked yeast concentration (cells/mL)	CV% flow counts	CV% plate counts
3 × 10^1^	8.462	41.807
3 × 10^2^	7.899	19.080
3 × 10^3^	7.139	7.699
3 × 10^4^	7.700	7.041
3 × 10^5^	4.958	7.025
3 × 10^6^	2.590	5.162

## 4 Discussion

Using a novel blood culture processing workflow and unique combination of fluorescent markers, we have developed a rapid flow cytometric method capable of detecting clinically important yeast (including *C. auris*) with results available in just over 1 h. Commercially available fungal stains such as CFW have been used in fluorescence microscopy in clinical laboratories for decades (Hageage and Harrington, 1984). CFW binds to β-1-3 and β-1-4 polysaccharides found in both cellulose and chitin in yeast (Wood and Fulcher, 1983) and has been previously used to examine chitin content in pure fungal cultures using flow cytometry (Costa-de-Oliveira et al., 2013). The use of CFW, ACF, and ConA to detect yeast in high priority clinical specimens (such as blood cultures) using flow cytometry has not been performed in earlier studies. In our study, we first demonstrated highly accurate enumeration of yeast cells across all tested species in pure cultures (stained with CFW, Supplementary Figure 1) to prepare standardized suspensions. Unlike CFW which binds to specific compounds, the surface antigens or epitopes that ACF binds to are not specifically listed in the literature. We found that there was minimal staining of CFW and ACF to CD235a and CD45 positive cells, in contrast to ConA which had high non-specific staining. ConA is a lectin that binds to mannoproteins in the yeast cell wall (Biely et al., 1976), however studies have also found that human cells such as erythrocytes and polymorphonuclear leucocytes can express glycoproteins which act as a binding site for the lectin (Albertini et al., 1977; Okada, 1981; Schmalstieg et al., 1985). This was consistent with the staining phenotype we observed and additional validation was performed using confocal microscopy. ConA was observed to stain additional non-yeast cells (Figure 6) and to improve assay specificity, we selected CFW and ACF for our staining panel.

In our study, we observed differences in the CFW staining profile across the yeast species tested. Differences in CFW fluorescence have been reported in an earlier study (Costa-de-Oliveira et al., 2013) and these differences have been associated with variations in chitin and glucan content in the fungal cell wall (de Groot et al., 2008). The combination of both CFW and EB staining enabled us to achieve consistent separation of yeast cells from background noise (human cells/debris) after blood culture clean-up, particularly in low CFW staining yeast such as *N. glabratus*. In addition, the use of the YER gate demonstrated high specificity for yeast cells (94.67%) as validated using the Attune^TM^ Cyptix^TM^ images. EB is included in the CFW solution as a counterstain to quench background fluorescence of other tissues and cells (Hageage and Harrington, 1984; Lynch and Gibson, 1987; Harrington and Hageage, 2003). The dye is membrane impermeable (Hamer et al., 2002; Smith et al., 2015), has been used as a viability stain in plant cell studies (Vijayaraghavareddy et al., 2017) and as an indicator for compromised human cell membranes (Miller et al., 2007). As our blood processing clean-up method uses both Triton-X to denature membranes (Jamur and Oliver, 2010) and sterile water to induce osmotic lysis, we hypothesized the human cell populations would be damaged while the yeast cells would be largely intact due to the presence of rigid cell walls which protect cells from osmotic stress (Smith et al., 2000; Levin, 2011). This hypothesis was supported using imaging cytometry as the population staining higher in EB and lower in CFW (background noise region) consisted of human cells with various morphotypes including bits of debris (Figures 4A, B). Human cells were also observed to be adhered to yeast cell aggregates which could account for the higher EB staining phenotype seen in the cell yeast population (Figure 4D). Recent studies showing increased adhesion and biofilm formation in clinical isolates of *C. albicans* (de Camargo Ribeiro et al., 2025) provide a plausible biological basis for this observation and speak to the potential for this method as part of investigations of fungal virulence.

Given bacterial blood-stream infections are more common than episodes caused by yeast (Chiarini et al., 2008; Chmielarczyk et al., 2021), we investigated the possibility of non-specific staining of our selected markers for both Gram-negative and positive species. We focused on the most common bacterial organisms isolated from positive blood cultures, including *E. coli, S. aureus, S. epidermidis, K. pneumoniae, and P. aeruginosa* (Chua et al., 2023; Holmes et al., 2025). We did not observe any non-specific staining of CFW in the gram-negative isolates tested (Figure 7) and this finding is consistent with the structure of Gram-negatives as the thin peptidoglycan layer sits below the outer-membrane (Beveridge, 1999; Silhavy et al., 2010). In contrast non-specific staining was observed in both *S. aureus* and *S. epidermidis* (Figure 8) as the thick peptidoglycan layer in gram-positives consists of alternating β-1,4-linked *N*-acetylglucosamine and *N-*acetylmuramic acid (Rohde, 2019). Previous studies using fluorescence microscopy have also made this observation as CFW labeled the biofilms of *S. aureus, S. epidermidis* (Myllymaa et al., 2013), and *H. influenzae* (Domenech et al., 2016) (with the matrix composed of β-linked glycans).The use of the second fluorescent marker (ACF) was a vital component in our assay design to ensure that true candidemia was not obscured by the presence of Gram-positive bacterial organisms in positive blood cultures.

This study demonstrates the capability of flow cytometry to provide rapid and accurate detection/enumeration of yeast cells from both spiked and clinical blood cultures. In spiked blood cultures, flow cytometry demonstrated superior precision to plate counts across the dilutions tested (Table 2) and a strong correlation (linear regression, *R*^2^ = 0.934) to plate counts at the countable range (Figure 10). Our flow cytometric assay for detection was more sensitive compared to conventional methods for detection such as Gram-staining which has an LOD of 10^5^ CFU/mL for yeasts (using machine-assisted interpretation) (Walter et al., 2024) and peripheral blood smears with detection ranging from 10^5^ to 10^7^ CFU/mL (Branda et al., 2007). Compared to microscopy, the increased sensitivity in our assay could translate to detecting candidemia at an earlier stage (prior to flagging positive in automated systems) and/or detecting low yeast cell concentrations in samples with slow growing yeast species. While the LOD of our assay in its current stage of development may not be as sensitive as genotypic methods for detection such as PCR based techniques (such as the T2Candida test with a reported LOD of 1–3 CFU/mL) (Mylonakis et al., 2015), our phenotypic based assay could be used in combination with other rapid methods. In addition, the quantitative data provided by flow cytometry could be utilized for rapid antifungal susceptibility testing (AST) from blood cultures and potentially provide AST results days earlier than conventional methods. For applications where greater sensitivity is required, our method could be further optimized: increasing the volume of specimen tested and correlating quantitative measurements from multiple serial dilutions are both avenues to increasing the statistical power of rare event detection. An additional advantage of our study was the direct application of the assay to yeast-positive clinical blood cultures, reflecting “real-life” conditions. We were able to detect all yeast in the samples analyzed, including three new species (*S. cerevisiae, C. kefyr* and *C. lusitaniae)* that were previously not tested during assay optimization. While candidemia with species such as *S. cerevisiae* is rare, it still has been reported in a range of patient groups including immunocompromised patients, neonates and individuals in the ICU (Ipson and Blanco, 2001; Muñoz et al., 2005; Ventoulis et al., 2020) which make detection of these species still vital.

While our protocol can rapidly detect yeast cells in blood cultures, we acknowledge several limitations. Firstly, while we have examined the staining dynamics in bacterial spiked cultures, we acknowledge that this assay has not been extensively applied directly to clinical bacterial blood cultures. As our design may not exactly replicate the biological complexity and immunological host responses in our spiked samples, future studies are required to assess the performance of the assay in real-world bacterial specimens. Other factors affecting enumeration accuracy such as human-yeast aggregates and prior antifungal exposure will also need further investigation. In addition, the performance of the assay in poly-microbial cultures (yeast and bacteria) should be more thoroughly evaluated as one study reported synchronous bacteremia in 24% of candidemia cases (Klotz et al., 2007). Our study was also limited by small sample sizes and while we focused on the detection of yeasts, other important pathogens including filamentous fungi (such as *Aspergillus* sp.) and encapsulated yeasts (*Cryptococcus* sp.) need to be considered as mortality rates associated with these fungal infections can exceed ~43% (Dao et al., 2024; Denning, 2024). While previous studies have stained these pathogens with CFW (Zhang et al., 2024; Upadhya et al., 2023), for universal applicability to different fungal species, the assay would need additional optimization. In particular, the processing and staining protocol we developed for blood cultures would need to be optimized for different biological matrices as these pathogens can be isolated from cerebral spinal fluid and bronchoalveolar lavage samples.

## 5 Conclusion

Our study presents a unique and rapid approach of detecting and enumerating yeast species in blood cultures with results provided in just over 1 h. Using a panel of selected markers, we validated our gating strategy and demonstrated the robustness of the assay when applied directly to clinical blood culture samples. With setting-specific optimization, flow cytometry shows promise as a reliable method for the rapid detection of candidemia, which could be integrated into a clinical diagnostic pathway for serious fungal infections.

## Data Availability

The raw data supporting the conclusions of this article will be made available by the authors, without undue reservation.
