# Supplementary material for: Fast-tracking fungal detection: a rapid flow cytometric method for the detection of yeasts in blood cultures
Source: Front Microbiol. 2026 Apr 23;17:1813126. doi: 10.3389/fmicb.2026.1813126 (PMC13149291; doi:10.3389/fmicb.2026.1813126)
Supplement: Supplementary file 1 [file Table_1.docx]

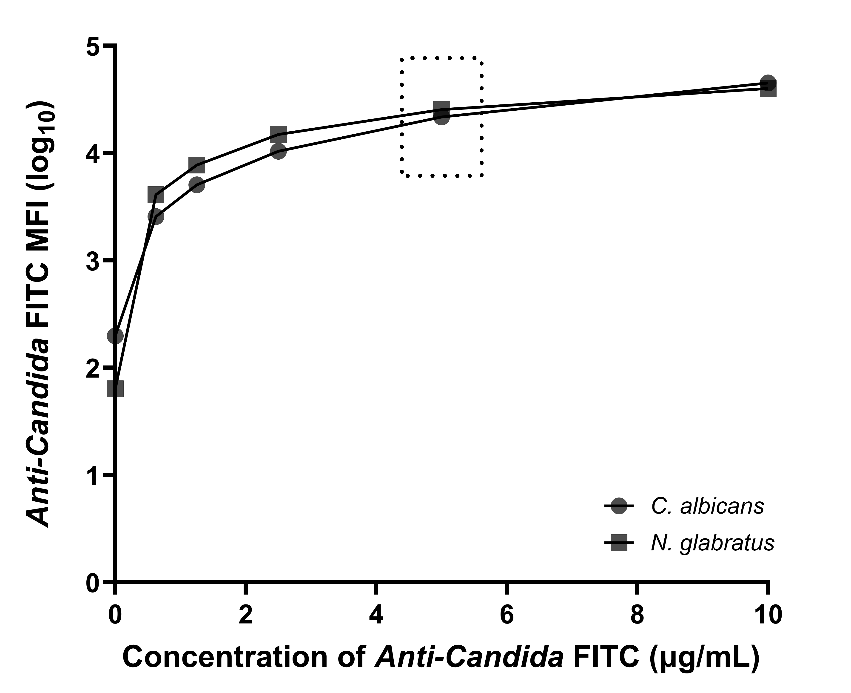

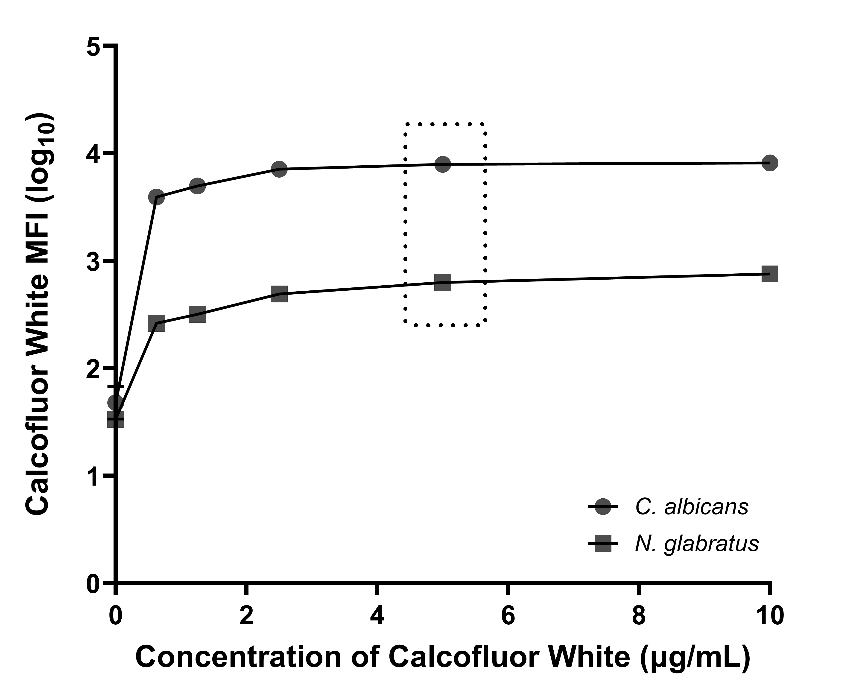
Supplementary Material

**B**

**A**


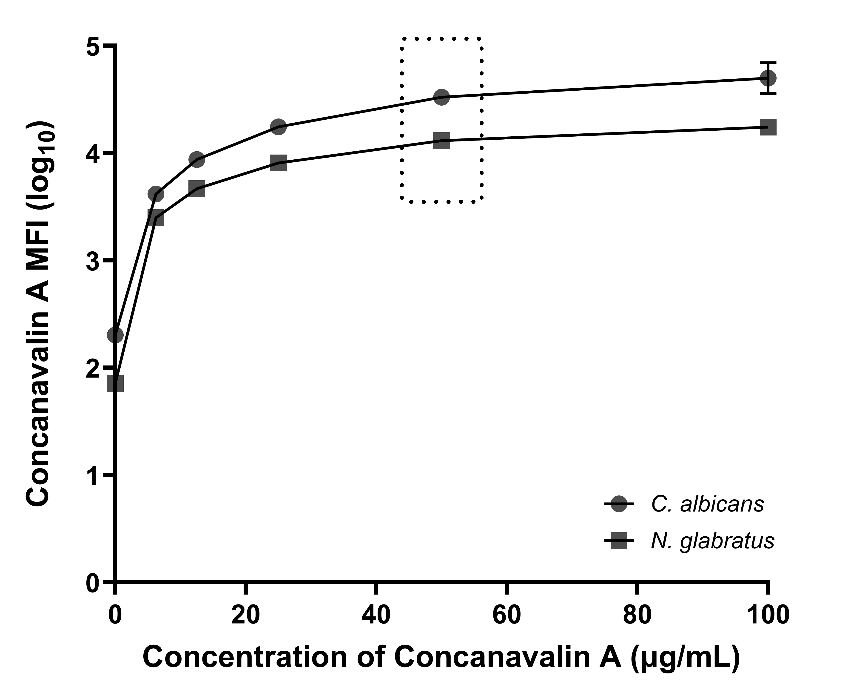


**C**

**Supplementary Figure 1.** **Yeast marker titration curve for Calcofluor White (A), Anti-*Candida* FITC and Concanavalin A (C), tested using *C. albicans* and *N. glabratus* (n = 2 isolates).** The median fluorescence intensity (log_10_ transformed) for each species is plotted against the concentration of stain. For Anti-*Candida* FITC concentrations, the highest concentration for each tested range is displayed (e.g. 4 – 5 µg/mL shown as 5 µg/mL). Data is plotted as mean with error bars depicting the SD. The optimal concentration for each stain is shown in the figure as a dashed box.


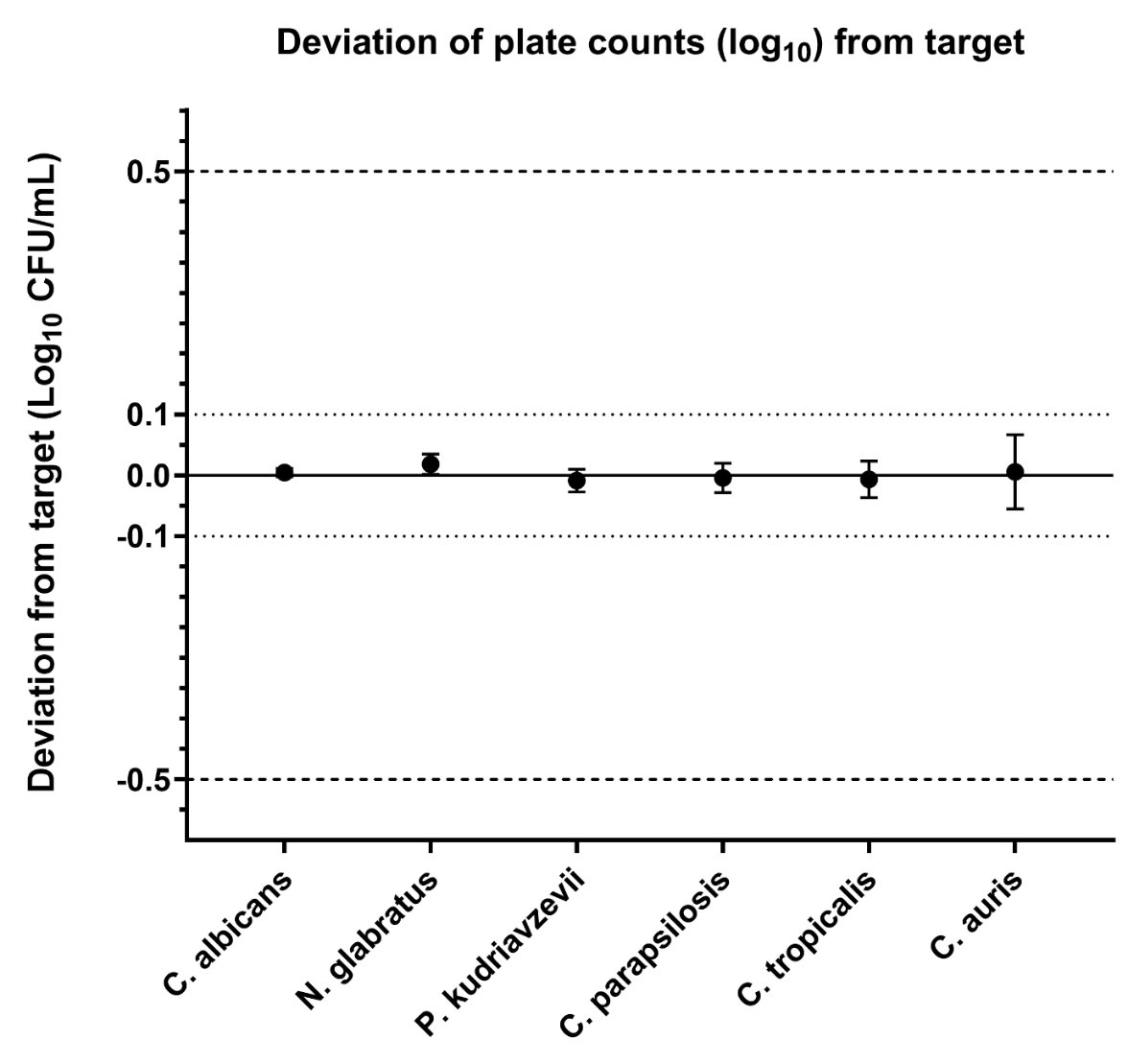


**Supplementary Figure 2.** **Flow cytometric enumeration of yeast cells is highly accurate in pure cultures.** The mean deviation from the target suspension (5 log_10_ CFU/mL – depicted as the solid line) is shown for isolates prepared for standardised spiked blood cultures (n = 29 total). Error bars show SD. Dotted lines show a ±0.1 and ±0.5 log_10_ deviation from the target suspension.
